# Supplementary material for: ISWI remodeler facilitates cBAF genomic binding to drive cell fate transition
Source: bioRxiv. 2026 May 12:2026.05.08.723508. Preprint. [Version 1] doi: 10.64898/2026.05.08.723508 (PMC13192760; doi:10.64898/2026.05.08.723508)
Supplement: Supplement 1 — Figure 2-S1. Snf2h is required for myogenesis and adipogenesis in culture (A-B) Snf2h is required for myogenesis in culture. Muscle satellite cells isolated from Snf2hf/f;CreER mice were treated with 4OHT, followed by myogenesis assays. (A) Representative microscopic pictures of satellite cells at D3 of myogenesis. (B) qRT-PCR analysis of myocyte marker genes Myog, Myh1, and Ckm during myogenesis. (C-E) Snf2h is required for adipogenesis in culture. SV40T-immortalized Snf2hf/f;CreER preadipocytes were treated with 4OHT, followed by adipogenesis assays. (C) Western blot analysis of Snf2h in preadipocytes. RbBP5 was used as a loading control. (D) Deletion of Snf2h does not affect cell growth rates. 1 x 10^5^ preadipocytes were plated and cumulative cell numbers were determined for 5 days. (E) Oil Red O staining at D7 of adipogenesis. (F-I) RNA-Seq analysis before (D-3) and during (D2) adipogenesis. (F) Expression of Snf2h and Snf2l at D-3 and D2. RPKM values indicate gene expression levels. (G) Genome browser view of Snf2h (upper panel) and Snf2l (lower panel) loci at D-3 and D2. (H) Pie chart depicts Snf2h-dependent and - independent up-regulated genes as well as down-regulated genes from D-3 to D2 of adipogenesis. The cut-off for differential expression is 2-fold. (I) Gene ontology (GO) analysis of 618 Snf2h-dependent and 1735 Snf2h-independent up-regulated genes defined in (H). Figure 2-S2. Snf2h depletion prevents myogenesis and adipogenesis in culture (A-C) C2C12 cells were infected with lentiviral vector expressing control (Ctrl) or Snf2h knockdown shRNA (shSnf2h), followed by myogenesis assays. (A) Western blot analysis of Snf2h in C2C12 cells. (B) Representative microscopic pictures at D5 of myogenesis. (C) qRT-PCR analysis of myocyte marker genes Myog, Myh1, and Ckm during myogenesis. (D-F) Myogenesis in CRISPR/Cas9-mediated Snf2h KO C2C12 cells. (D) Schematic of generating Snf2h KO C2C12 cells using a gRNA. (E) Western blot analysis of Snf2h and MyoD protein l [file NIHPP2026.05.08.723508v1-supplement-1.pdf]

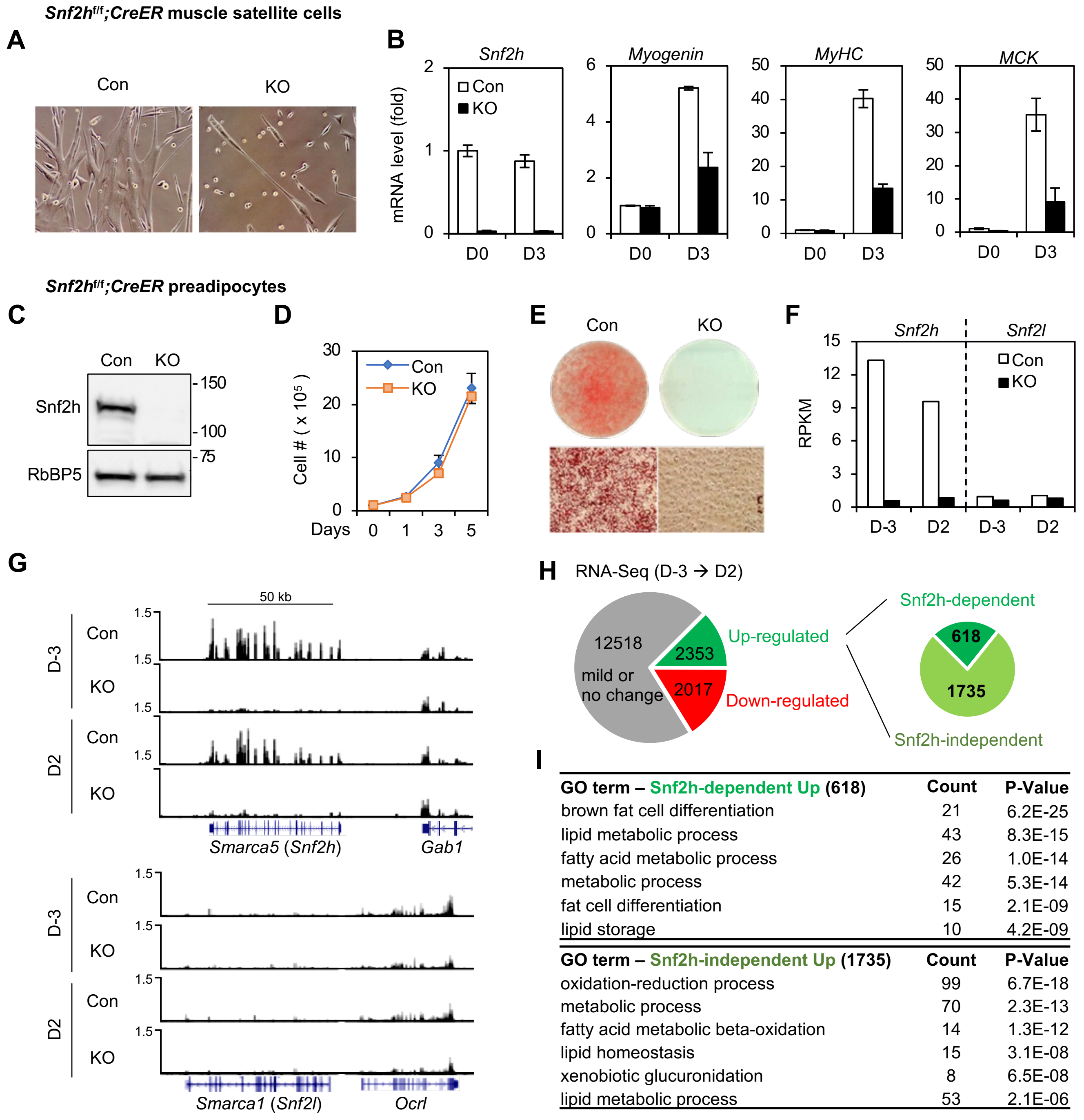

GO term – Snf2h-dependent Up (618)	Count	P-Value
brown fat cell differentiation	21	6.2E-25
lipid metabolic process	43	8.3E-15
fatty acid metabolic process	26	1.0E-14
metabolic process	42	5.3E-14
fat cell differentiation	15	2.1E-09
lipid storage	10	4.2E-09

GO term – Snf2h-independent Up (1735)	Count	P-Value
oxidation-reduction process	99	6.7E-18
metabolic process	70	2.3E-13
fatty acid metabolic beta-oxidation	14	1.3E-12
lipid homeostasis	15	3.1E-08
xenobiotic glucuronidation	8	6.5E-08
lipid metabolic process	53	2.1E-06

Figure 2-S1. Snf2h is required for myogenesis and adipogenesis in culture

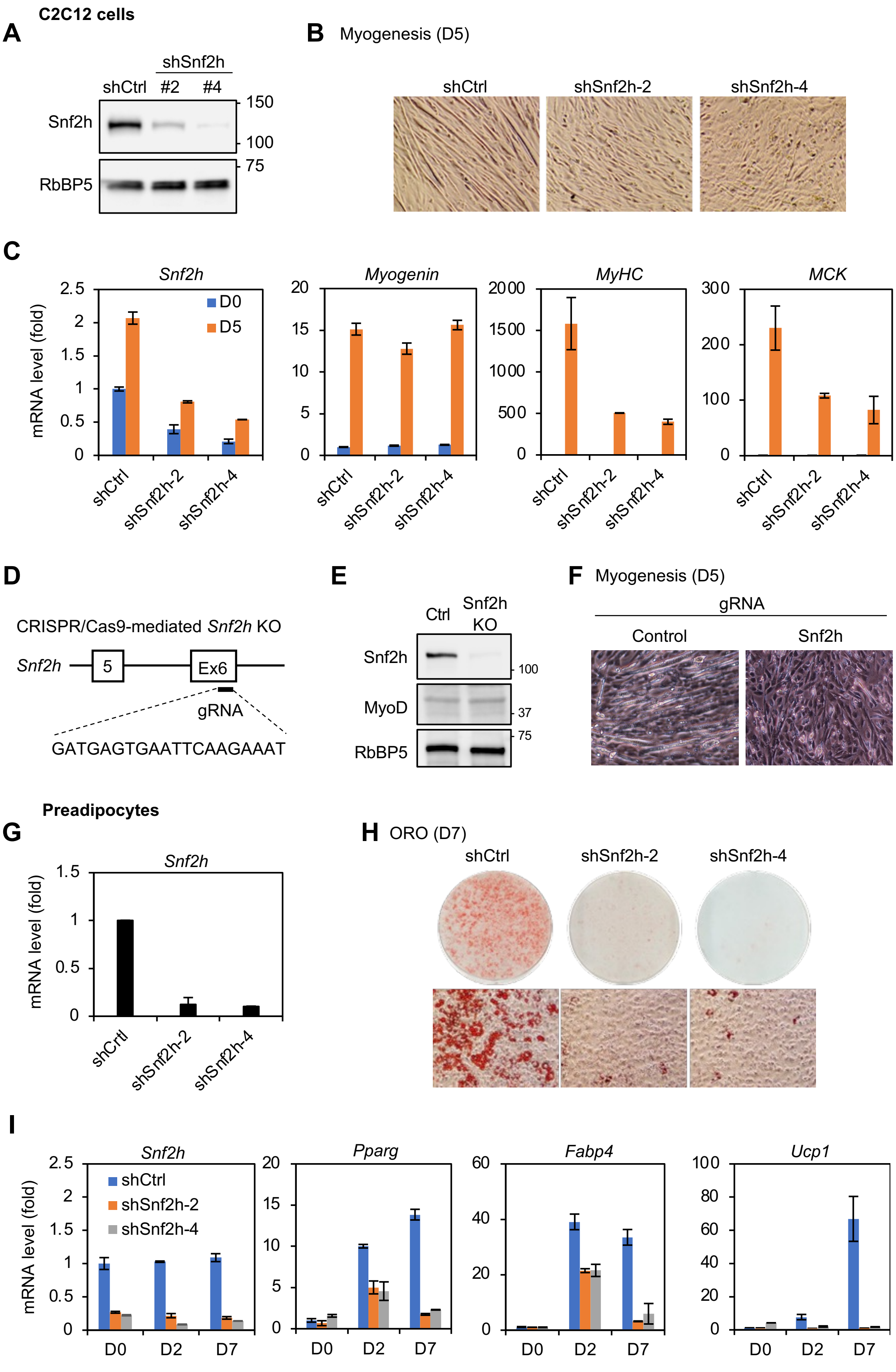

Figure 2-S2. Snf2h depletion prevents myogenesis and adipogenesis in culture

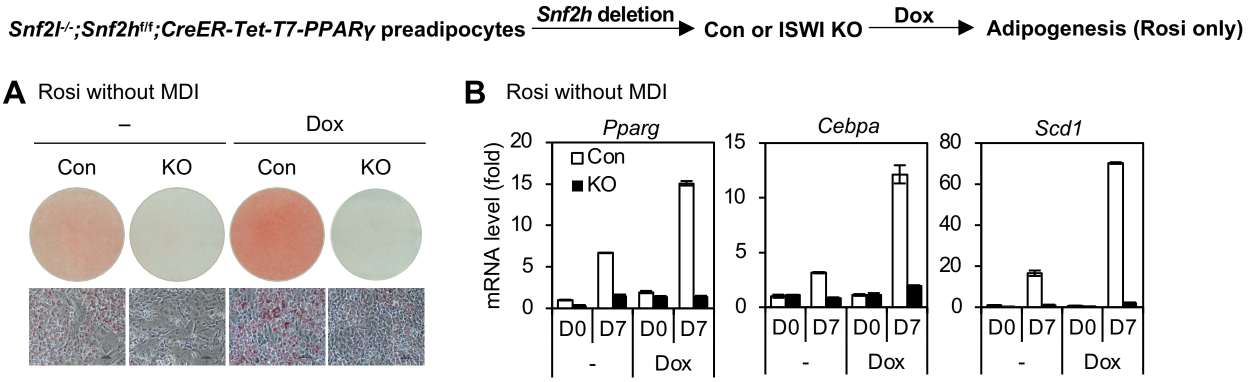

Figure 3-S1. Stable knockout of ISWI prevents PPARγ ligand-stimulated adipogenesis

***Snf2l*$^{-/-}$;*Snf2h*$^{f/f}$;*CreER-Tet-MyoD-T7* preadipocytes** —*Snf2h* **deletion**→ **Con or ISWI KO** —**Dox, 1 day**→ **WB, ChIP-Seq**

A GREAT GO analysis

MyoD binding in ISWI KO	GO term	P value
Intact	regulation of leukocyte differentiation	3E-63
	regulation of hemopoiesis	4E-60
	regulation of angiogenesis	1E-58
	regulation of apoptotic signaling pathway	4E-49
	regulation of protein ser/thr kinase activity	6E-38
	regulation of smooth muscle cell migration	5E-34
>2-fold decreased	response to insulin stimulus	2E-45
	myeloid leukocyte differentiation	1E-26
	generation of precursor metabolites & energy	1E-24
	PDGFR signaling pathway	1E-22
	myotube differentiation	2E-17
	collagen fibril organization	3E-17

B Genes associated with ISWI-dependent MyoD binding

GO term	Genes
myotube differentiation	*Actn3, Adgrb1, Ankrd2, Bhlhe41, Cflar, Csrp3, Cxcl12, Cxcl9, Cyp26b1, Dmpk, Ehd1, Ehd2, Gdf15, Hdac4, Hdac5, Hdac9, Il1f9, Il4ra, Lmod3, Maml1, Mapk14, Myf5, Myf6, Myocd, Myog, Nfatc2, Nkx2-5, Nln, Notch1, Nov, Rbm24, Rbm38, Ripor2, Scgb3a1, Shox2, Sik1, Smyd1, Tbx1, Thra, Trim72*

C

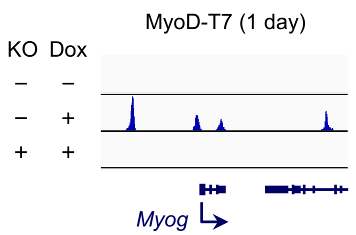

D

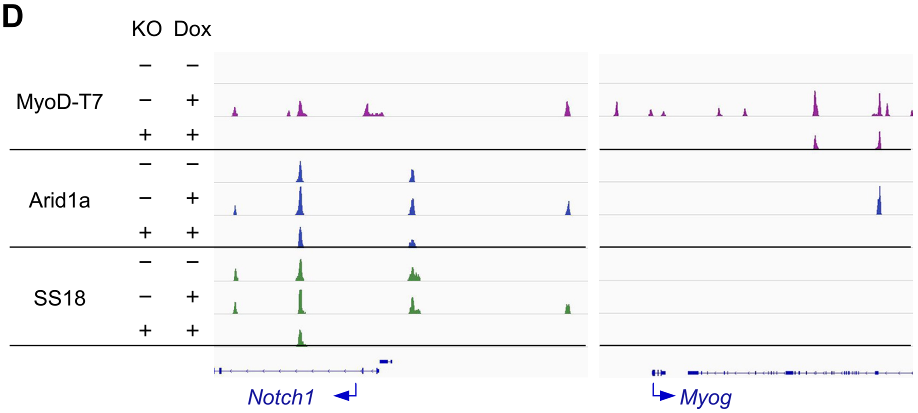

Figure 4-S1. Stable knockout of ISWI disrupts *de novo* binding of MyoD and cBAF on chromatin

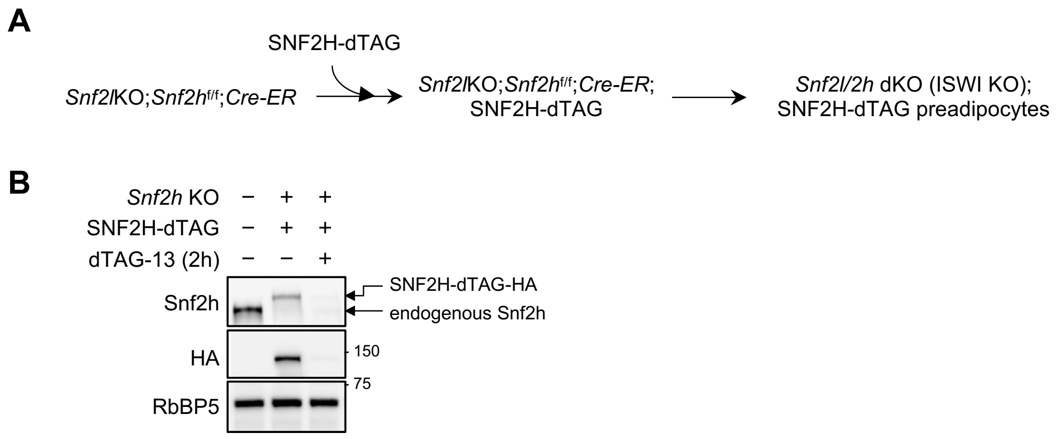

Figure 5-S1. Generation of cell line for acute SNF2H depletion using dTAG system

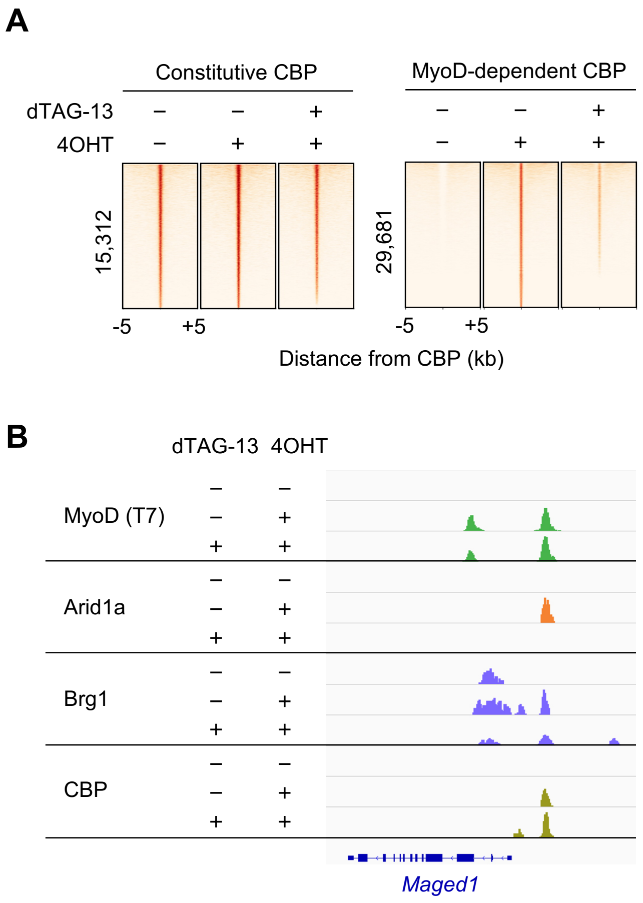

Figure 6-S1. Acute depletion of ISWI disrupts *de novo* cBAF binding
